# The primary stability of ultrashort residual proximal femur fixed with triangular fixation stem prosthesis: a comparative biomechanical study based on sawbones models

**DOI:** 10.3389/fbioe.2024.1493738

**Published:** 2024-10-14

**Authors:** Ziwei Hou, Kai Zheng, Ming Xu, Xiuchun Yu

**Affiliations:** Department of Orthopedics, The 960th Hospital of the People’s Liberation Army, Jinan, China

**Keywords:** proximal femur, prosthesis, stem, biomechanical study, sawbones

## Abstract

**Background:**

Tumor resection near the proximal end of the femur and revision surgery of the distal femoral prosthesis may result in a very short bone segment remaining at the proximal end of the femur, known as ultrashort residual proximal femur (URPF). In this study, we propose a triangular fixation stem (TFS) prosthesis to improve the fixation of URPF. The aim of this research is to investigate the biomechanical properties of the TFS prosthesis and compare it with the conventional stem (CS) prosthesis through *in vitro* biomechanical experiments, providing preliminary biomechanical evidence for prosthetic fixation of URPF.

**Methods:**

A biomechanical study was conducted using Sawbones to explore initial stability. Twelve Sawbones were used to create a bone defect model, and prostheses were designed and fabricated to emulate TFS fixation and CS fixation structures. Axial compression and horizontal torsion experiments were performed on the fixed models using a mechanical testing machine, recording maximum displacement, maximum torque, and femoral strain conditions.

**Results:**

Under an axial compressive load of 2800 N, the overall displacement of the TFS group was 3.33 ± 0.58 mm, which was significantly smaller than that of the CS group (4.03 ± 0.32 mm, P = 0.029). The femoral samples of the TFS group demonstrated that the strain value alterations at the medial points 2, 3, 5, 6 and the lateral point 10 were conspicuously smaller than those of the conventional stem group (P < 0.05). Under torsional loads at levels of 1°, 3°, and 5°, the torques of the TFS group were 3.86 ± 0.69 Nm, 3.90 ± 1.26 Nm, and 4.39 ± 1.67 Nm respectively, all of which were significantly greater than those of the CS group (1.82 ± 0.82 Nm, P < 0.001; 2.05 ± 0.89 Nm, P = 0.016; 1.96 ± 0.50 Nm, P = 0.015 respectively).

**Conclusion:**

The TFS prosthesis improves fixation strength and reduces strain on the femur’s proximal surface. Compared to CS fixation, it offers better resistance to compression and rotation, as well as improved initial stability.

## 1 Introduction

The femur is a frequent location for malignant bone tumors among the long bones of the limbs (Ma et al., 2019). With the increasing application of tumor prostheses in the reconstruction of femoral defects, numerous orthopedic oncologists have discovered that extensive resection of tumor adjacent to the femoral epiphysis (particularly the proximal femur) might lead to a residual bone segment that is overly short to permit the implantation of a conventional prosthetic stem (Christ et al., 2021a; Dieckmann et al., 2014; Shehadeh et al., 2019). Similarly, such problems may arise during revision surgery (Min et al., 2018; Zimel et al., 2016). Bone loss resulting from complications such as aseptic loosening and infection can also give rise to residual short bone segments. This presents a significant challenge for limb salvage treatment of bone tumors. Tsukamoto et al. (2023) initially brought up ultrashort residual proximal femur (URPF), and they held the view that bone defects with a length of less than 12 cm should be fixed by specialized prostheses. The correlation between the existing reconstruction approaches and postoperative function, as well as the risk of complications, was summarized.

The reconstruction of URPF poses a highly challenging problem for bone oncologists. Due to the short residual bone segment and the specific anatomical and biomechanical features of the proximal femur, reconstruction with conventional prostheses will inevitably encounter a high risk of aseptic loosening (Greig et al., 2021; Wang et al., 2021). Current reconstruction methods include Compress Compliant Prestress (CPS) implants (Avedian et al., 2014; Christ et al., 2021b), Allograft-prosthetic composite (APC) replacement (Hindiskere et al., 2021; Moon et al., 2013), personalized short stems such as Buxtehude stems (Cannon et al., 2003) and interlocking reconstruction stem-lateral plate implants (Christ et al., 2021a). Although these methods attain initial fixation in the early and mid-terms, mechanical complications like prosthesis or screw breakage and prosthesis loosening still arise. This might be closely associated with the prosthesis design not conforming to the biomechanical characteristics of the proximal femur.

It is widely acknowledged that the bone trabeculae in the proximal femur are not randomly arranged but are orderly disposed in accordance with the force direction. The tension and pressure trabeculae form Ward’s triangle and calcar at the center of the intersection of the femoral neck, jointly constituting an essential structure for mechanical conduction in the proximal femur. Based on this biomechanical property, some orthopedic surgeons have enhanced the traditional proximal femoral intramedullary nail fixation system and achieved favorable clinical efficacy as well as robust biomechanical evidence (Ding et al., 2022). To explore a more stable URPF reconstruction scheme, we put forward a prosthesis design featuring a triangular fixation stem (TFS) to enhance URPF fixation. Theoretically, TFS fixation is stabler than traditional conventional stem (CS) fixation. To our best knowledge, there are no biomechanical studies regarding prosthetic fixation of URPF. In this study, the fixation stability of TFS and CS was compared through *in vitro* biomechanical experiments.

## 2 Materials and methods

A total of 12 Sawbones femur models (#3406, left, large, Pacific Laboratories, United States) belonging to the same production batch were used for this study.

### 2.1 Design and fabrication of prosthesis

**FIGURE 1:**
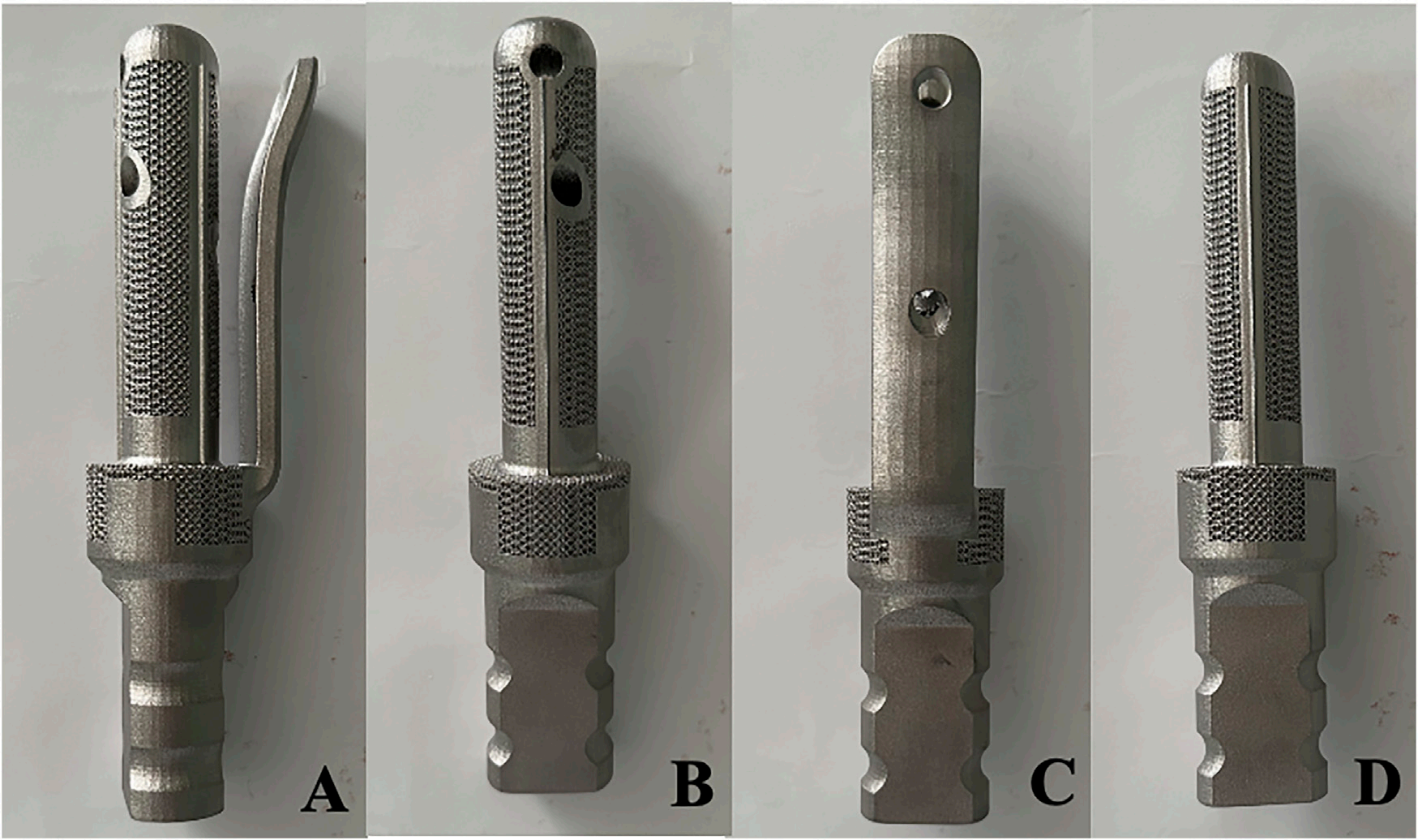
Photos of the appearance of TFS and CS prostheses **(A–C)** Front view and internal and external views of the TFS prosthesis; **(D)** Front view of the CS prosthesis.

TFS and CS were customised from Sawbones CT data. The stem had a diameter of 18 mm and a length of 90 mm. The TFS comprised a custom-made stem featuring a lateral plate and locking screws. Two 5 mm locking screws were placed in the metaphyseal bone in a cross distribution through the stem. The lateral plate was fixed to the lateral side of the proximal femur by means of locking screws. The bone-bone interface of the stem was a porous metal structure (depth 3 mm, thickness 3 mm, diameter 400 μm, porosity 70%). The bottom end of both stems was a conical structure that could be connected to a conventional modular prosthesis. However, for suitability for the clamping of the mechanical testing machine, we transformed the bottom end into a cylindrical shape to facilitate the embedding and fixation of the denture powder. After the design was accomplished, the titanium alloy was fabricated through selective laser melting technology and 3D printing. The follow-up encompassed a series of treatments such as drilling, tapping and polishing (Figure 1).

### 2.2 Osteotomy and fixation

The URPF bone defect model was established by conducting an osteotomy 12 cm beneath the horizontal line of the apex of the femoral head, referring to the literature of Tsukamoto et al. (2023). Following the resection of the femoral model, the diameter of the medullary cavity was measured at 16 mm, with a depth of 50 mm. To accurately simulate the prosthesis placement during surgery and in accordance with preoperative surgical planning, we employed a mechanical reamer to modify the femoral model. Ultimately, twelve femoral models were produced, each featuring a diameter of 17 mm and a length of 90 mm. Prior to implanting the 3D-printed metal stem, trial implantation was conducted using a resin material stem. If compatibility between the stem and medullary cavity was confirmed, the resin stem was removed; debris within the medullary cavity was subsequently cleared before securing the femoral model onto tiger table forceps. TFS and CS were then implanted into the femoral marrow cavity along its longitudinal axis, ensuring careful verification of both prosthesis positioning and integrity of the femoral model.

**FIGURE 2:**
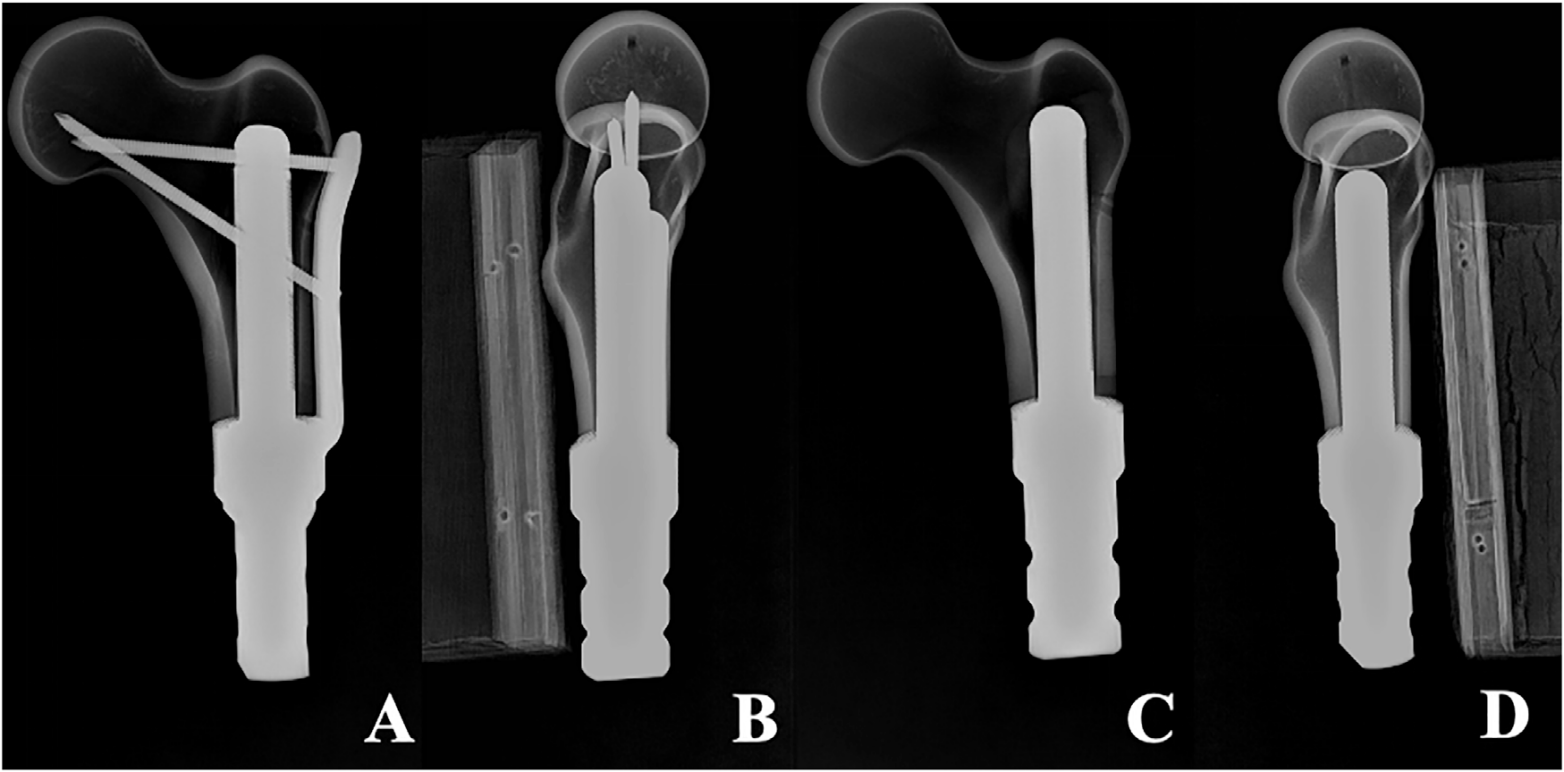
X-ray of two models **(A, B)** Anterolateral radiograph of Femur-TFS **(C, D)** Anterolateral radiograph of femur-CS.

Once the fixation was completed, the position of the prosthesis and whether the femoral specimen was cracked were meticulously examined. Subsequently, drilling, tapping, and screwing of the corresponding locking screw were carried out. Eventually, six femoral-TFS models, designated as the TFS group, and six femoral-CS models, designated as the CS group, were acquired. X-ray were taken to observe the position of the prosthesis and screws, as well as whether Sawbones had latent fractures (Figure 2). All of these establish uniform and standardized conditions for biomechanical comparisons.

### 2.3 Biomechanical tests

**FIGURE 3:**
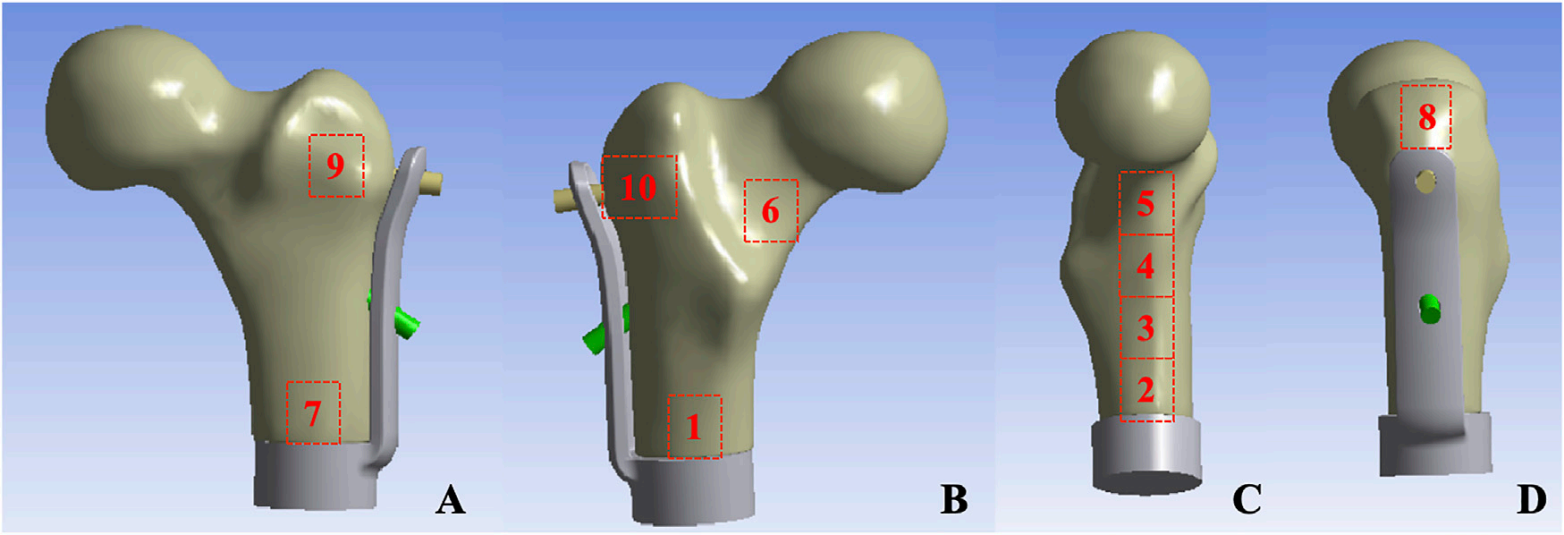
Femur surface strain value measurement location: **(A)** Anterior aspect of femur **(B)** Posterior aspect of femur **(C)** Medial aspect of femur **(D)** Lateral aspect of femur.

Mechanical testing was conducted utilizing an ElectroForce 3510 mechanical testing machine (Bose, United States). Prior to the test, the base of the model was embedded with denture powder, ensuring that the model was maintained in the frontal plane of 15° adduction and the sagittal plane of 5°–10° vertical and internal rotation (Bong et al., 2004), in order to simulate the normal human lower limb force line. Resistance strain gauges were pasted at ten sites (numbered 1–10) on the surface of the femur to obtain the stress changes on the surface of the femur (Figure 3). The bottom end of the model was firmly fixed using a self-made distal femoral fixture and subsequently connected to a mechanical testing machine. To simulate the stress environment of the hip joint accurately, a self-made acetabulum (Polymethyl Methacrylate material) was placed on the femoral head during the axial compression test. The compression device of the testing machine exerted downward pressure on the acetabulum to guarantee that the acetabulum was in close proximity to the femoral head. During the horizontal torsion test, the self-made proximal femoral clamp was linked to the compression device, and the clamp was attached to the femoral head, enabling the top of the femoral head to rotate internally only, without any lateral movement. Ensure that the model remains in a single-legged standing position throughout.

#### 2.3.1 Axial compression test

**FIGURE 4:**
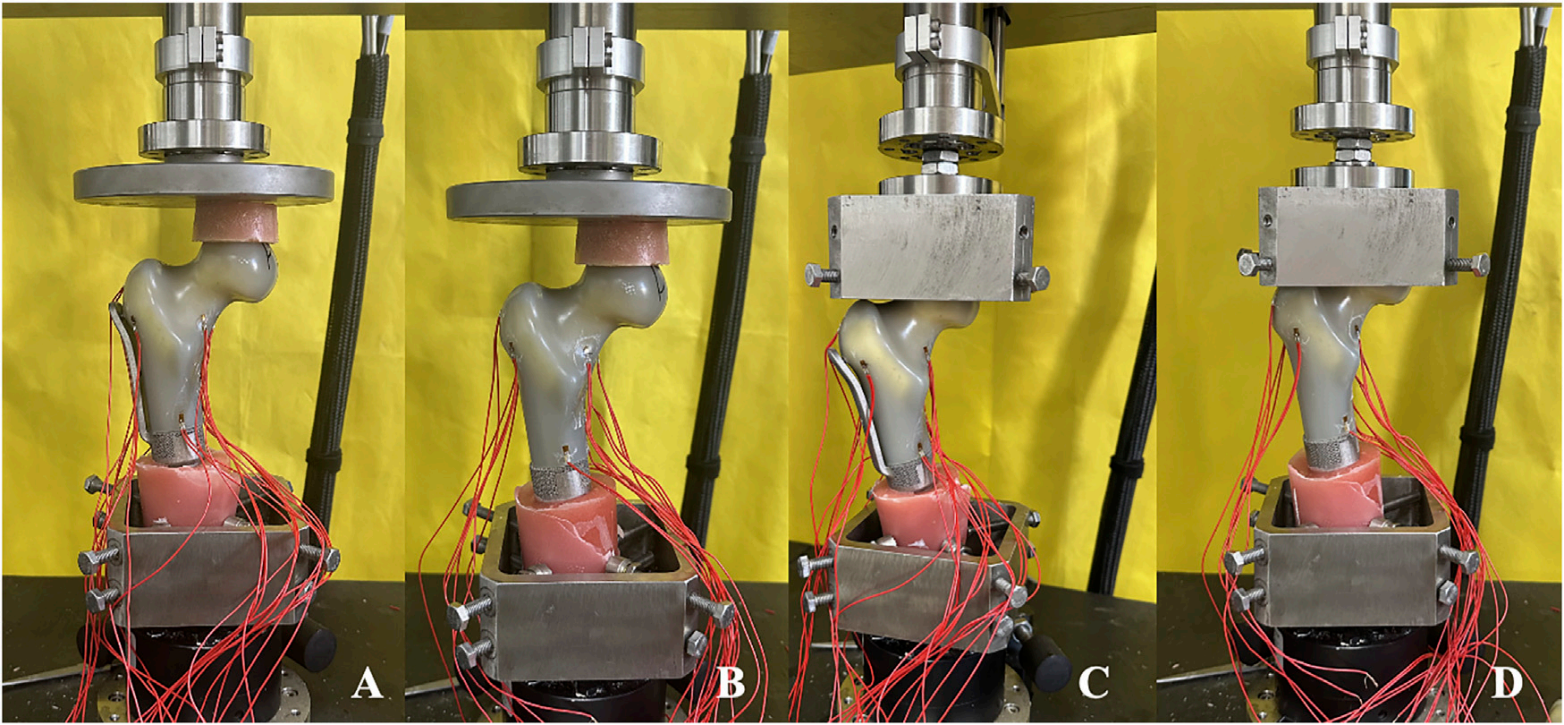
Axial compression and horizonal torsion tests of two groups of models **(A, C)** TFS **(B, D)** CS.

The model was affixed to testing machine and preloaded with an axial compression load of 100 N for 1 min to ensure closer contact between the femoral head and acetabulum and to mitigate the creep effect of the model. After initializing the testing machine and the strain gauge system software, the axial dynamic load ranged from 0 to 2,800 N (equivalent to four times the body weight of a 70 kg adult) at a speed of 10 mm/min. The displacement data corresponding to the load and the strain value of ten strain gauges were recorded throughout the test. Each sample was tested three times, and the average of the data from the three tests was taken as the final data. Each experiment was spaced 30 min apart to enable the model to fully return to its initial state (Figure 4).

#### 2.3.2 Horizontal torsion test

The model at the conclusion of the axial compression test was fastened on the self-made proximal femoral clamp. During the fixation process, caution was exercised to avoid gripping the femoral head overly tightly to prevent an excessive initial torque resulting from overly tight fixation. Once the model was fixed, the horizontal torsion test perpendicular to the force line was conducted, and the direction of torsion was the external rotation of the proximal femur. The initial preload was 2 Nm and lasted for 30 s, enabling the force line testing machine, the self-made fixture, and the model to be closely combined and reducing the creep. After resetting the test software of the mechanical testing machine to zero, the torsion angle was loaded from 0° to 5° at a rate of 0.05 rad/s, and the angle and corresponding torque were recorded. Each sample was tested three times, and the average of the data from the three tests was taken as the final data. Each experiment was spaced 30 min apart to permit the model to fully return to its initial state (Figure 4).

### 2.4 Data processing and statistical approaches

The load and displacement in the axial compression test, as well as the torque and torsion angle in the horizontal torsion test, were directly measured by the sensors of the mechanical testing machine and recorded at a frequency of 20 Hz on a dedicated data acquisition computer. The strain test system recorded the measured strain values at a frequency of 20 Hz. The data obtained in this experiment were processed by SPSS 20.0 statistical software and expressed in the form of mean ± standard deviation. For the data conforming to normal distribution, the difference between groups was analyzed by independent t-test, while for the data not conforming to normal distribution, nonparametric test was employed. P < 0.05 was regarded as statistically significant.

## 3 Results

### 3.1 Axial compression test

**FIGURE 5:**
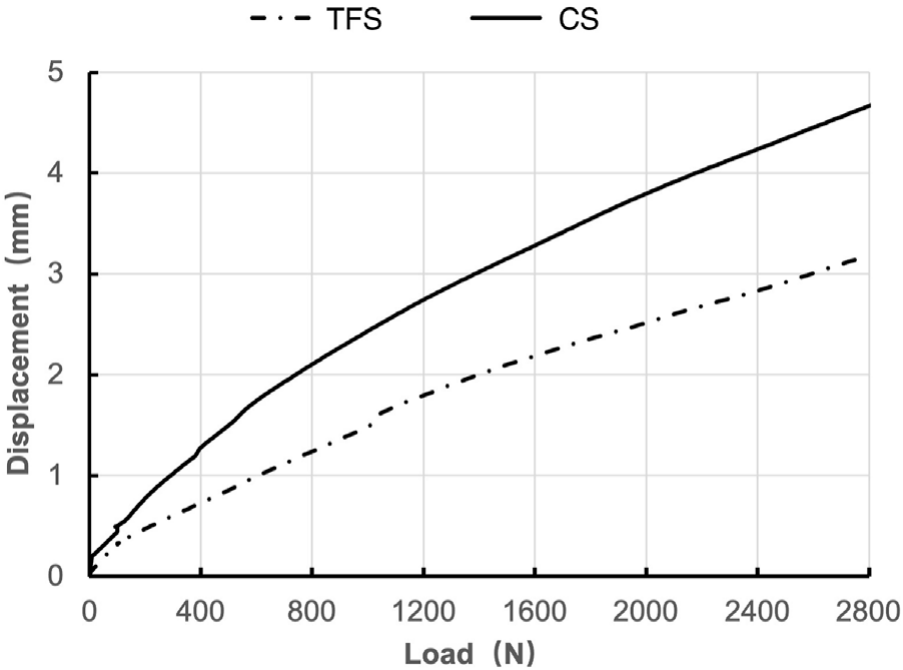
Load-displacement graphs of TFS and CS.

**FIGURE 6:**
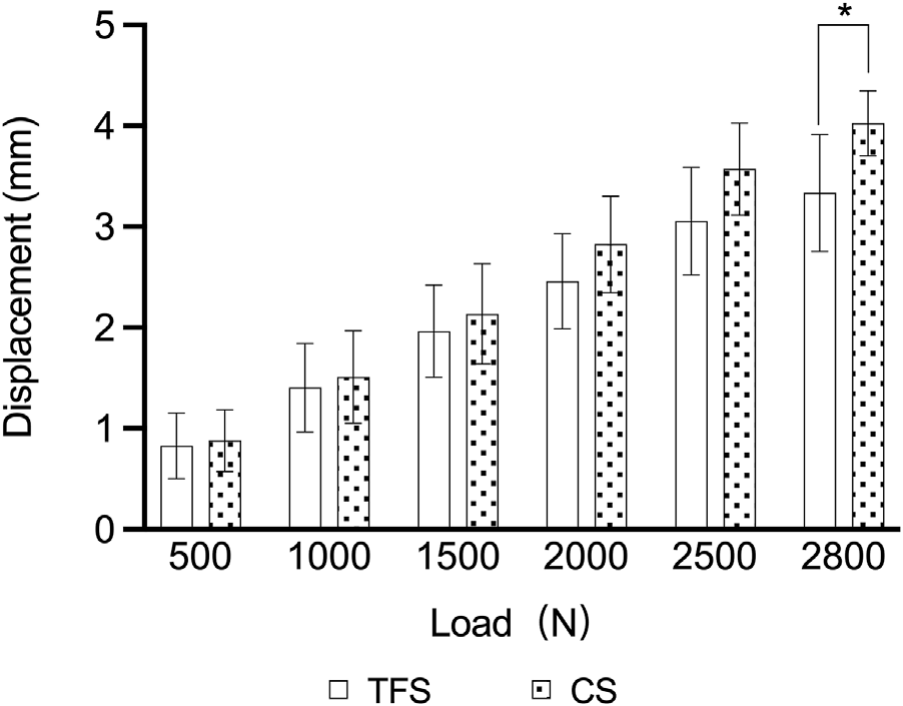
Comparison of the load-displacement outcomes of the two groups of models* = p < 0.05.

During the loading process ranging from 0 to 2800 N, the load-displacement curves of the two groups of models were approximately straight lines (Figure 5), which was in accordance with the linear variation, indicating that the specimens of the two groups underwent elastic deformation. The experimental results demonstrated that under an axial compression load of 2800 N, the displacement of group TFS (3.33 ± 0.58 mm) was significantly smaller than that of group CS (4.03 ± 0.32 mm, t = 2.556, P = 0.029) (Table 1, Figure 6).

**TABLE 1:** Comparison of the load-displacement outcomes of the two groups of models.

Load (N)	Displacement (mm)		t	*P*
	Group TFS [mean (SD)]	Group CS [mean (SD)]		
500	0.83 (0.32)	0.88 (0.31)	0.29	0.778
1,000	1.41 (0.44)	1.51 (0.46)	0.405	0.694
1,500	1.96 (0.45)	2.13 (0.49)	0.63	0.543
2000	2.45 (0.46)	2.82 (0.47)	1.338	0.105
2,500	3.05 (0.53)	3.57 (0.45)	1.802	0.103
2,800	3.33 (0.58)	4.03 (0.32)	2.556	0.029

The measurement results of the femoral surface strain value indicated that compressive stress was borne on the medial side of the lesser trochanter and tensile stress was borne on the lateral side, which was in accordance with the biomechanical distribution direction of the femur. The results demonstrated that the force direction of the ten points was identical, with compressive stress at points 2, 3, 4, 5, 6, and 7, and tensile stress at points 8, 9, and 10. The strain values of group TFS at points 2, 3, 5, 6, and 10 were significantly lower than those of group CS (−618.08 ± 231.95 vs. −1777.14 ± 709.77, P = 0.003; −1,509.66 ± 537.01 vs. −2,174.79 ± 406.94, P = 0.036; −2,241.19 ± 276.04 vs. −2,789.73 ± 264.34, P = 0.019; −1,177.29 ± 269.49 vs. −1849.91 ± 452.14, P = 0.017; 583.56 ± 369.52 vs. 1,321.06 ± 796.13, P = 0.012). There was no significant difference in model strain values between the two groups at points 1, 4, 7, 8, and 9 (P > 0.05) (Table 2).

**TABLE 2:** Comparison of the value of strain of the two groups of models.

No.	Value of strain		t	*P*
	Group TFS [mean (SD)]	Group CS [mean (SD)]		
1	−192.15 (113.81)	−71.31 (35.73)	1.321	0.143
2	−618.08 (231.95)	−1777.14 (709.77)	3.802	0.003
3	−1,509.66 (537.01)	−2,174.79 (406.94)	2.418	0.036
4	−1,599.23 (257.33)	−1826.27 (425.41)	1.119	0.289
5	−2,241.19 (276.04)	−2,789.73 (264.34)	3.035	0.019
6	−1,177.29 (269.49)	−1849.91 (452.14)	2.901	0.017
7	−612.72 (448.58)	−153.81 (40.99)	1.358	0.175
8	223.03 (68.70)	240.91 (139.26)	0.282	0.784
9	311.91 (96.49)	553.38 (145.7)	1.623	0.136
10	583.56 (369.52)	1,321.06 (796.13)	1.594	0.012

### 3.2 Horizontal torsion test

**FIGURE 7:**
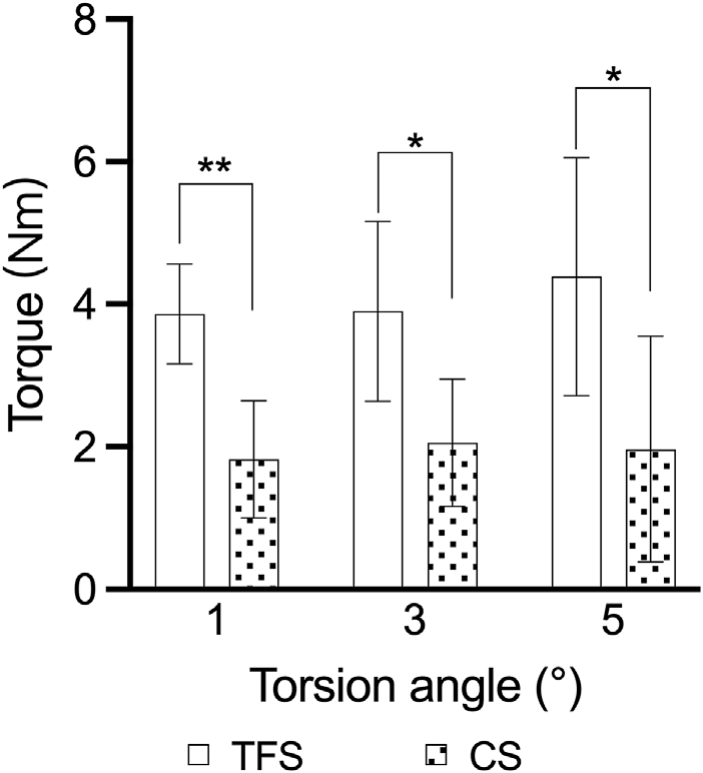
Comparison of the torsion angle-torque outcomes of the two groups of models * = p < 0.05, ** = p < 0.01.

The outcomes of the horizontal torsion test indicated that the torque of group A was conspicuously higher than that of group B at 1°, 3° and 5° torsion (3.86 ± 0.69 vs. 1.82 ± 0.82, P < 0.001; 3.90 ± 1.26 vs. 2.05 ± 0.89, P = 0.016; 4.39 ± 1.67 vs. 1.96 ± 0.50, P = 0.015) (Table 3, Figure 7).

**TABLE 3:** Comparison of the torsion angle-torque outcomes of the two groups of models.

Torsion angle (°)	Torque (Nm)		t	*P*
	Group TFS [mean (SD)]	Group CS [mean (SD)]		
1	3.86 (0.69)	1.82 (0.82)	4.637	< 0.001
3	3.90 (1.26)	2.05 (0.89)	2.911	0.016
5	4.39 (1.67)	1.96 (0.50)	3.103	0.015

## 4 Discussion

For URPF, the application of CS fixation is bound to augment the risk of aseptic loosening of the prosthesis. Fuchs et al. demonstrated that the implantation of a standard stem necessitated adequate cortical bone mass and a medullary cavity of at least 5 cm for accommodation, and regarded the length of the medullary cavity less than 5 cm as a contraindication for the implantation of a standard prosthetic stem (Fuchs et al., 2008). Streitbürger et al. (2022) indicated that the metaphyseal and diaphyseal regions of the femur were most prone to aseptic loosening (39.1%) in a follow-up of 28 patients with bone tumor resection and reconstruction using a segmental prosthesis. Owing to the limited bone/cement interface, CS fixation is unable to withstand the intense pressure and rotational forces caused by hip motion. Currently, approaches to enhance URPF fixation comprise the addition of lateral auxiliary cortical plates (Christ et al., 2021a; Stevenson et al., 2017), cross-cross screw fixation (Dieckmann et al., 2014; Cannon et al., 2003; Bernthal et al., 2019), and surface treatment (You et al., 2022). Even though these methods enhance certain stability, there are still issues such as prosthesis loosening and screw breakage.

The biomechanical properties of the proximal femur are highly correlated with its unique anatomical structure. In 1838, Ward initially proposed the concept of “Ward’s triangle,” that is, the pressure trabecular system and the tension trabecular system of the proximal femur form a distinct Trigonum at the central area of the femoral neck intersection, namely Ward’s triangle. According to Wolff’s law (Wolf, 1995), the anatomical structure of bone is in alignment with its function. During the transmission of gravitational load in the proximal femur, the force direction will be dispersed to the pressure bone trabecula and the tension bone trabecula, and its trend is approximately triangular. Based on this characteristic, we proposed a TFS to enhance the fixation of URPF. Its design concept mainly encompasses two points: (1) The utilization of triangularly distributed cross screws for assisting fixation; (2) Fixation with a lateral plate. To verify the superior biomechanical properties of the TFS, we compared the overall stability of the TFS with that of the CS *in vitro* mechanical experiments. The results of this experiment indicated that the TFS was more stable than the CS fixation.

The results of the axial compression test reveal that the overall displacement of TFS is conspicuously smaller than that of CS when the load attains 2800 N, suggesting that the utilization of TFS demonstrates superior axial compression resistance. Our analysis indicates that this is closely associated with the screw distribution that aligns with the direction of mechanical conduction in the proximal femur and the lateral plate. Firstly, among the two screws in triangular distribution, the horizontal support screw can disseminate the lateral tensile stress, and the transcervical fixation screw can convey the medial compressive stress. The two screws can effectively disperse the stress on the hip joint during weight-bearing and walking, and prevent the stress concentration phenomenon. The study by Nie et al. (2020) demonstrated that the application of Medial sustainable nail with triangular structure exhibits better stability than the common PFNA. Secondly, the design of the lateral plate not only shares the force transmission but also effectively enhances the fixation strength. Huang et al. (2022), in their biomechanical study on the fixation of femoral metaphphysis with a new axial compression internal prosthesis, indicated that the stem + lateral plate design possesses better stability than the simple stem.

The outcomes of the horizontal torsion test demonstrated that the torque of TFS was conspicuously greater than that of CS at 1°, 3°, and 5° of horizontal torsion, signifying that TFS possessed superior anti-rotation capacity. The hip joint is exposed to high magnitudes of combined axial, bending, and torsional loads during activities of daily living. Research have indicated that the torsion torque of the hip joint can attain a maximum of 37 Nm when the patient stands on one foot after total hip arthroplasty (Heinecke et al., 2018). For URPF, the restricted femoral marrow cavity and a considerable amount of cancellous bone render the stem of the prosthesis highly prone to rotation after implantation. Prior studies have revealed that diverse stem designs, encompassing stem diameter, length, shape, structure, and surface, are crucial factors influencing the rotational stability of proximal femoral prostheses (Meneghini et al., 2006; Holsgrove et al., 2013). The lateral plate and two triangular fixation screws of TFS undoubtedly augment the overall anti-rotation ability, enabling a greater torque to be acquired at 5° of horizontal torsion.

The results of femoral surface strain indicated that the strain distribution of the femur in the CS group was non-uniform, with the maximum strain value being −2,789.73 ± 264.34 and the minimum strain value being −71.31 ± 35.73. Under such a condition, a portion of the cortex endured a considerable force, which was disadvantageous for the integration of the implant-bone interface. The maximum strain of TFS was −2,241.19 ± 276.49, and the minimum strain was −192.15 ± 113.81. At points 2, 3, 5, and 6 of the medial femoral regions, the strain of the TFS group was smaller than that of the CS group. This implies that the screws in TFS close to the medial femoral cortex can effectively disperse the compressive stress of the surrounding area. For the lateral region around the greater trochanter, tensile stress was detected at sites 8, 9, and 10, and the strain values in the TFS group were lower than those in the CS group, and the difference at site 10 was statistically significant (583.56 ± 369.52 vs. 1,321.06 ± 796.13, P = 0.012). This might be associated with the fact that the lateral plate and the screw close to the greater trochanter bear the tensile stress of the area around the nail hole. Frost (2004) hold that appropriate strain stimulation is conducive to bone formation. Thus, TFS can bear more loads than CS to restore the normal mechanical conduction characteristics of the proximal femur, which is beneficial for the interface integration between the prosthesis and bone after implantation. Therefore, based on the disparity in strain value changes between the two groups, we contend that TFS can better disperse the stress concentration on the femoral surface than CS, prevent stress shielding, and possess better stability.

This study is subject to certain limitations. Firstly, the sample size is relatively small, and only a set of control groups was established without an appropriate blank control. This may introduce biases due to uncontrollable factors such as experimental equipment and environmental conditions. Secondly, the mechanical loading applied by the testing machine may not accurately replicate the magnitude and direction of loads experienced by the femur under normal physiological conditions. Furthermore, this study did not account for soft tissue structures such as muscles, ligaments, and joint capsules; thus, the findings may diverge from the actual biomechanical properties observed in human anatomy.

In conclusion, TFS not only enhances the stability of the stem structure *per se*, but also effectively mitigates the strain on the proximal femoral surface. In comparison with CS, TFS demonstrated superior resistance to compression and rotation, as well as better stability. From a biomechanical perspective, TFS constitutes a rational scheme for URPF reconstruction, providing robust biomechanical evidence and support for subsequent clinical application.

## Data Availability

The raw data supporting the conclusions of this article will be made available by the authors, without undue reservation.
